# Evaluation of the Adjuvant Effect of Dexmedetomidine on Ropivacaine for Transversus Abdominis Plane Block in Inguinal Hernia Repair: A Prospective Double-Blind Randomized Trial

**DOI:** 10.3390/jcm14072478

**Published:** 2025-04-04

**Authors:** Kassiani Theodoraki, Ioannis Koutalas, Christina Orfanou

**Affiliations:** Department of Anesthesiology, Aretaieion University Hospital, National and Kapodistrian University of Athens, 11528 Athens, Greece; ktlsjohn@gmail.com (I.K.); chrisorf@hotmail.com (C.O.)

**Keywords:** hernia, inguinal, analgesia, regional, peripheral nerve block, analgesia, postoperative, pain, acute, pain, chronic, local anesthetic, ropivacaine, dexmedetomidine, adjuvant

## Abstract

**Background and goal of study:** The aim of this double-blind randomized study was to investigate the efficacy of dexmedetomidine as an adjuvant to the local anesthetic in transversus abdominis plane (TAP) block for unilateral inguinal hernioplasty. **Materials and Methods:** Eighty eligible patients were randomly allocated into ultrasound-guided TAP block with either dexmedetomidine 0.5 mcg/kg diluted to a volume of 2 mL and ropivacaine 0.5% 25 mL (DR group) or ropivacaine 0.5% 25 mL and normal saline 2 mL (R group). The primary endpoint of this study was the numeric rating scale (NRS) score during coughing 24 h postoperatively. Secondary parameters were also evaluated. **Results:** Patients in the RD group demonstrated significantly less pain at rest three, six and 12 h postoperatively as compared to patients in the R group (*p* = 0.002, 0.032 and 0.049, respectively). Significant differences between the two groups were also demonstrated for NRS scores during coughing at 3, 6 and 12 h postoperatively (*p* = 0.013, 0.035 and 0.042, respectively). Additionally, the RD group demonstrated lower intraoperative remifentanil consumption (*p* < 0.001), lower PACU morphine requirement (*p* = 0.012) and lower overall PCA morphine requirement postoperatively (*p* < 0.001). Sedation scores, the incidence of hypotension, bradycardia and the occurrence of postoperative nausea and vomiting were no different between the two groups. Finally, the incidence of chronic pain at 6 months was significantly lower in the RD group compared to the R group (5.55% vs. 25%, *p* = 0.049). **Conclusions:** Dexmedetomidine as an adjuvant to ropivacaine reduces postoperative pain scores, has opioid-sparing effects and is associated with a favorable effect on chronic pain without side effects in patients subjected to TAP block for inguinal hernia repair.

## 1. Introduction

Inguinal hernia repair is a relatively simple surgical procedure, affecting both men and women and being most commonly performed in the day-case setting. One of the most popular techniques for inguinal hernia repair is the Lichtenstein “tension-free” hernioplasty using mesh prostheses, which is considered the standard of care for unilateral and bilateral hernia repair and is accompanied by a low recurrence rate. Despite the simplicity and the short operative time of the Lichtenstein repair technique, inguinal hernioplasty can occasionally lead to moderate or severe postoperative pain, which can unduly prolong hospitalization and affect functional status post-surgery or delay return to normal daily activities. Furthermore, inadequate management of postoperative pain may lead to the development of chronic pain. Chronic pain after inguinal hernia repair is a well-recognized entity with a reported prevalence occasionally exceeding 30% [1,2]. It can have both neuropathic and nociceptive features and may untowardly affect overall quality of life as well as create psychological imbalance in involved individuals. Apart from inadequate management of acute postoperative pain, other predisposing factors involved in the development of chronic pain post-inguinal hernia repair are the existence of pain in the inguinal area before surgery and nerve injury during the operation [3].

In recent years, regional anesthetic techniques have been successfully used as adjuncts to systemic analgesia, aiming at minimizing abundant opioid use. One of these regional techniques that has been increasingly utilized in a variety of upper and lower abdominal surgery procedures as part of a multimodal analgesic regimen is the transversus abdominis plane (TAP) block. Studies have documented its effectiveness in reducing postoperative pain intensity, time to first rescue analgesic request and overall opioid demand after surgery [4,5,6,7]. The TAP block is a field block technique, which involves local anesthetic deposition in the neurofascial plane between the internal oblique and transversus abdominis muscles. The aim is to inhibit nociceptive signals emanating from the anterolateral abdominal wall and traveling via the anterior divisions of spinal nerves T7 to L1, which are present in this plane as the lower intercostal, ilioinguinal and iliohypogastric nerves [8]. Although the TAP block was initially developed as a blind landmark-guided technique, in recent years, harnessing the popularity of ultrasound-assisted regional blocks, it has almost exclusively been performed as an ultrasound-guided technique. Due to its technical simplicity and the small learning curve, ultrasound-guided TAP block has been increasingly utilized for pain control as part of a multimodal analgesic approach in a variety of surgical procedures, both open and laparoscopic [9,10,11,12]. Accordingly, studies have emerged that support the use of ultrasound-guided TAP block as useful analgesic adjunct in inguinal hernia repair procedures [13,14,15].

Ropivacaine is a local anesthetic widely used in perineural block techniques due to its prolonged action. Further prolongation of the blockade afforded by ropivacaine is not always feasible due to the fear of local anesthetic-associated potential toxicity. In the quest for improvement of the effect and duration of TAP block, the use of various adjuvants such as magnesium sulfate, dexamethasone and buprenorphine has been advocated as an addition to the local anesthetic in order to prolong its analgesic effects [16,17].

Dexmedetomidine is a highly selective α-2 adrenoceptor agonist, which can block sympathetic nervous activity and provide anxiolysis, sedation and analgesia [18]. Apart from reducing analgesic requirements during general anesthesia, dexmedetomidine has also been effectively used as an adjuvant to the local anesthetic mixture in central and peripheral nerve blocks [19,20,21]. Apart from augmentation of analgesic action, there may also be additional interactions between dexmedetomidine and local anesthetics, which further support dexmedetomidine use as an adjuvant. For instance, dexmedetomidine may reduce the convulsive effects of simultaneously administered local anesthetics [22]. There is also experimental evidence of dexmedetomidine-afforded neuroprotection and reduction in local anesthetic neurotoxicity when dexmedetomidine supplements the administration of local anesthetics. Neuroprotection can be mediated via regulation of mast cell degranulation, through reduction in caspace-3-dependent nerve cell apoptosis or via regulation of protein kinase C expression and glutamate release [23,24,25].

In the context of inguinal hernia repair, the superiority of the mixture of dexmedetomidine–local anesthetic versus the local anesthetic alone for TAP block has scarcely been investigated [26,27]. In fact, by performing an extensive literature search, we could not find any study reporting the use of dexmedetomidine as an adjunct to ropivacaine in the TAP block for inguinal hernia repair in the adult population.

We hypothesized that the mixture of dexmedetomidine and local anesthetic would be superior to the local anesthetic alone. Therefore, the aim of this prospective double-blind randomized study was to evaluate the effect of adding dexmedetomidine to ropivacaine during ultrasound-guided TAP block in inguinal hernia repair in adult patients. We sought to investigate any potential advantages of the combination on acute postoperative pain parameters as well as on the development of chronic pain.

## 2. Materials and Methods

### 2.1. Trial Design, Ethics and Participants

This was a randomized double-blind placebo-controlled trial conducted between April 2018 and March 2024. All patients participating in the study provided written informed consent regarding anesthesia, invasive procedures involved and all study requirements. After evaluation of eligible patients during the preoperative visit by the anesthesiologist, 80 adult American Society of Anesthesiologists (ASA) I-II patients scheduled for elective unilateral inguinal hernia repair with a mesh under the Lichtenstein technique were enrolled in the study. Exclusion criteria were age under 18 years old and over 80 years old, ASA classification ≥ ΙΙΙ, body mass index over 35 kg/m^2^, reoperation of recurrent hernia after previous repair, skin infection around the needle insertion site, coagulation abnormalities, history of allergy to local anesthetics, known hepatic or renal impairment, contraindication to paracetamol or non-steroid anti-inflammatory drug administration and preoperative use of antidepressants, neuroleptics and opioids. Inability to consent to the study due to communication or language barriers or cognitive dysfunction precluding patient cooperation as to the precise evaluation of study endpoints were additional exclusion criteria.

Recruited participants were randomized with the help of a computer-generated sequence of random numbers via the use of online software (https://www.randomizer.org, accessed on 31 March 2018) into ultrasound-guided TAP block with either dexmedetomidine–ropivacaine (DR group) or ropivacaine alone (R group). Patients of the DR group were subjected to ultrasound-guided TAP block with ropivacaine 0.5% 25 mL and dexmedetomidine 0.5 mcg/kg diluted to a volume of 2 mL. Patients of the R group were subjected to ultrasound-guided TAP block with ropivacaine 0.5% 25 mL and normal saline 2 mL. Group allocations were kept in sequentially numbered opaque sealed envelopes, which were disclosed on the day of the operation by an anesthetic nurse upon patient arrival in the operating room to reveal which perineural solution would be administered. This anesthetic nurse prepared identical-appearing syringes of the solution intended for the TAP block and was not involved in further patient management. The attending anesthesiologist, the patient, the personnel involved in the patient’s postoperative care and the outcome assessor were all blinded to group allocation.

### 2.2. Anesthetic Procedure

During the preoperative visit, all patients were informed about the procedure and instructed as to the principle and use of the pain numeric rating scale (NRS), graded from 0 (zero pain) to 10 (worst possible pain), as well as to the use of a patient-controlled analgesia (PCA) device for postoperative management during the first 24 h post-surgery. In the operating room, standard monitoring consisting of pulse oximetry, three-lead electrocardiography and non-invasive blood pressure measuring was applied. After baseline hemodynamic data were noted, an indwelling intravenous catheter was secured. Subsequently, all patients were subjected to a standardized anesthesia regimen initiated with intravenous premedication with midazolam 0.025 mg/kg (Dormixal, DEMO A.B.E.E., Kryoneri, Greece), fentanyl 1 mcg/kg (Fentanyl Janssen, New Brunswick, NJ, USA), cimetidine 200 mg (Tagamet GlaxoSmithKlein, County Cork, Ireland) and metoclopramide 10 mg (Primperan Sanofi, Paris, France). Anesthesia induction was performed with propofol 2.5 mg/kg (Propofol-Lipuro 1%, B. Braun, Melsungen, Germany). After adequate preoxygenation, the patient’s airway was secured with a supraglottic airway device of the appropriate size for weight and gender (Supreme Laryngeal Mask, Malaysia, Teleflex Ireland). Afterwards, mechanical ventilatory support was initiated with intermittent positive-pressure ventilation with a 40% oxygen-in-air mixture and partial pressure of end-tidal carbon dioxide maintained at 30–35 mmHg. Anesthesia maintenance was accomplished with propofol 7 mg/kg/h and remifentanil between 0.1 and 0.5 mcg/kg/min (Dormiden, DEMO A.B.E.E., Greece) via electronic pump syringes. Remifentanil infusion was titrated intraoperatively so as to maintain mean arterial pressure and heart rate within 20% of baseline values.

After anesthetic induction, the unilateral TAP block was performed under real-time ultrasound guidance. The whole procedure took place under sterile conditions and after meticulous disinfection of the skin with a povidone–iodine solution. A portable ultrasound unit was used for the TAP block (Venue Go™, GE Healthcare, Chicago, IL, USA) via visualization with a linear array transducer probe with the frequency set at 10 MHz. The probe was positioned at the lateral part of the abdominal wall between the iliac crest and the subcostal margin at the umbilical level, maintaining a perpendicular orientation to an imaginary line joining the inferior rib and the anterior superior iliac spine. After careful manipulation of the probe, the three muscle layers of the lateral abdominal wall (external oblique, internal oblique and transversus abdominis muscle from the superficial to the deeper plane) were visualized (Supplementary Sonographic Video S1).

Followingly, a 22G short-beveled needle (StimuQuick Echo, Arrow International Inc, Brooklyn, OH, USA) was advanced using the in-plane insertion technique aiming at the aponeurosis between the internal oblique and transversus abdominis muscles, while care was taken to visualize the entire needle during advancement as a bright hyperechoic line. When the tip of the needle was visualized at the targeted fascial plane between the aponeuroses of the two muscles, the correct position of the needle was initially verified with the hydrodissection technique, which consisted of injection of 2 mL of normal saline solution. Followingly, the solution of either ropivacaine (Naropin-Ropivacaine HCL, Fresenius Kabi, Bad Homburg, Germany) and dexmedetomidine (Dexdor, Orion Pharma, Espoo, Finland) or ropivacaine alone according to group allocation was incrementally infused. Intermittent aspiration was performed throughout injection to ensure the avoidance of inadvertent intravascular administration of the local anesthetic solution, while correct needle placement was confirmed by visualization of local anesthetic deposition as an expanding hypoechoic elliptical shadow in the targeted plane between the aponeuroses of the internal oblique and transverse abdominis muscles. Only after 15 min had elapsed from completion of the TAP block with either ropivacaine and dexmedetomidine or ropivacaine alone was the surgical procedure allowed to start. To minimize confounding, all TAP blocks were performed by the same anesthesiologist, who was trained in ultrasound-guided techniques, while all surgical procedures were performed by the same two trained surgeons with the Lichtenstein mesh-based tension-free technique.

As part of standardized supplementary analgesia, all patients received paracetamol 1 g (Paracetamol Kabi, Fresenius Kabi, Germany) and parecoxib 40 mg (Dynastat Inj. Sol. Pfizer, Tadworth, Surrey, UK) approximately 30 min before the end of surgery. Intravenous administration of propofol and remifentanil was discontinued 10 min before the end of the operation, followed by patient emergence. After the patients were awake, breathing spontaneously and responding to verbal command, the laryngeal mask was removed. In the Post Anesthesia Care Unit (PACU), patients were administered 0.05 mg/kg morphine (Morfina Inj. Sol. IFET A.E., Pallini, Greece) bolus on request until the NRS score at rest was ≤3, and a lock-out period of 20 min was allowed before further bolus morphine administration. In case of postoperative nausea and vomiting (PONV), ondansetron 4 mg (Onda Vianex A.E., Pallini, Greece) was intravenously administered. Patients stayed in the PACU for at least one hour and were afterwards discharged as soon as they fulfilled the modified Aldrete criteria, provided that they had no PONV or an NRS score ≥4 on coughing. In the ward, all patients were administered a standardized analgesic regimen consisting of paracetamol 1 g every eight hours and parecoxib every 12 h. They also had access to a PCA device with a morphine solution made up to a concentration of 0.5 mg/mL of morphine to a total volume of 100 mL. The PCA device did not have a background infusion but had been set to administer 1 mg of bolus morphine as rescue analgesia with a 15 min lock-out period. A rescue dose of ondansetron was also administered in case of PONV.

### 2.3. Measurements and Study Endpoints

Pain was assessed during the early postoperative period, while the patients were still in the PACU, at 5 15, 30 and 60 min after the end of surgery with the NRS at rest. Pain was also assessed at three, six, 12 and 24 h postoperatively with the NRS at rest and on coughing. The incidence of hypotension and bradycardia intraoperatively and during PACU stay was also noted. Hypotension was defined as systolic arterial pressure <80% of baseline and bradycardia was defined as heart rate <50 beats per minute. Sedation level was also assessed 15 min after patient arrival to the PACU according to a four-point sedation score as follows: 1 for patient awake and alert, 2 for patient drowsy but responding to verbal commands, 3 for patient drowsy but arousable to physical stimuli and 4 for patient unarousable. Other parameters evaluated were the intraoperative dose of remifentanil infusion (mcg/kg), mg of morphine administered in the PACU, total dose of morphine administered via the PCA device during the first 24 h post-surgery, the incidence of PONV along with requests for ondansetron administration and any adverse effects associated with TAP block performance. Twenty-four hours postoperatively, patient satisfaction from postoperative analgesia on a four-point Likert scale, with 1 marked as dissatisfaction, 2 as neither satisfaction nor dissatisfaction, 3 as slight satisfaction and 4 as maximal satisfaction, was also assessed. Finally, patients were contacted at 6 and 12 months postoperatively by telephone for a brief interview to assess the presence of any residual pain or uncomfortable sensation at the operation site. The researcher that conducted the telephone interviews was blinded as to the group allocation.

### 2.4. Statistics

The primary endpoint of the study was the NRS score during coughing 24 h postoperatively. Sample size calculation was performed prior to the study and was based on the primary outcome and the information provided by a previous study from our group, which reported an NRS score during coughing 24 h postoperatively of 2.93 (SD 1.13) with a TAP block performed with a ropivacaine-only solution for inguinal hernia repair with the Lichtenstein technique [14]. By assuming that a 30% decrease in the NRS score with the use of ropivacaine in combination with dexmedetomidine would be clinically meaningful, we estimated that a sample size of 36 patients in each group would achieve a power of 0.90 and an alpha error of 0.05. We aimed for four more patients per group to allow for subject dropouts and any protocol violations. Parameters were tested for normality of distributions with the Kolmogorov–Smirnov test. Comparisons of numeric data between the two groups were performed with the unpaired *t*-test or the Mann–Whitney U-test for independent samples, depending on whether the variables followed a normal or non-normal distribution. The Chi-square or Fisher’s exact test as appropriate was used for comparisons of categorical data. NRS scores at rest and during coughing were analyzed with a two-factor mixed-design analysis of variance with repeated measures for one factor (time). The Student–Neuman–Keuls test was used post hoc for pairwise comparisons when appropriate. Results are expressed as mean (SD) or as median [25th–75th percentile], depending on the normality of distributions, and as absolute numbers (frequency) for categorical variables. A value of *p* < 0.05 was considered statistically significant. Data were analyzed with the SigmaPlot version 13 for Windows statistical software (Systat Software Inc., San Jose, CA, USA).

## 3. Results

**Figure 1:**
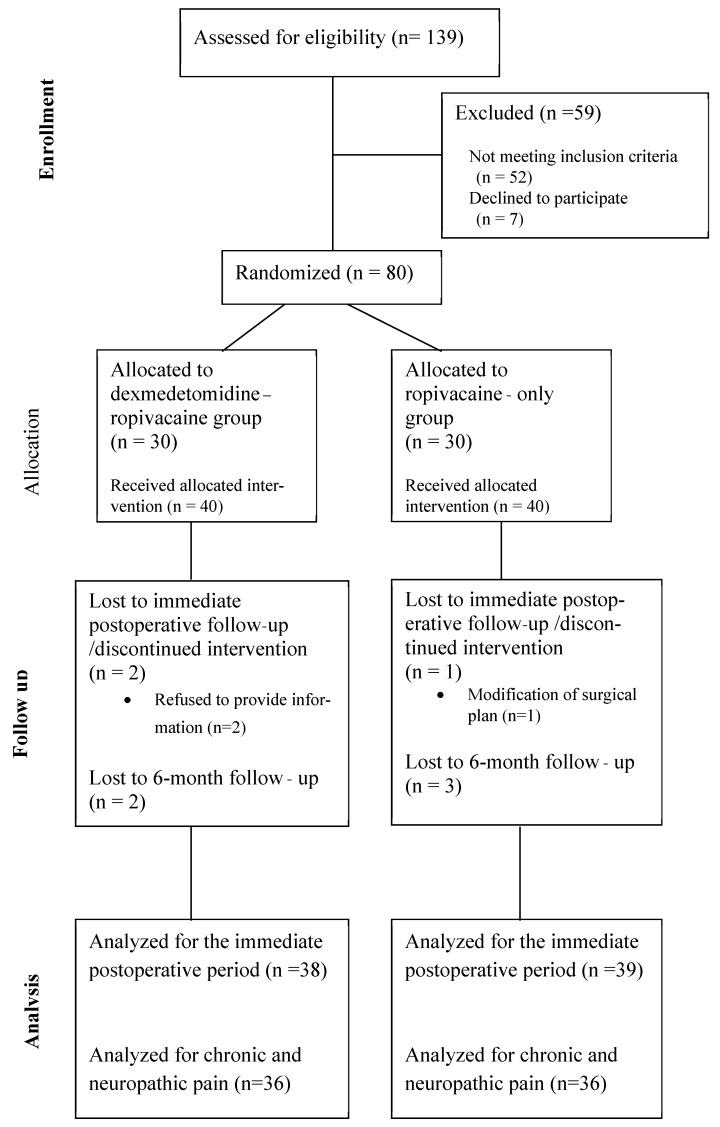
Study flowchart.

Throughout the study timeline, 139 patients scheduled for elective unilateral inguinal hernia repair with the Lichtenstein technique were approached and assessed for eligibility for the study. Among them, 80 patients fulfilled the criteria for participation and were recruited in the study. Forty patients were randomized to the dexmedetomidine–ropivacaine group and 40 to the ropivacaine-only group. Followingly, two patients in the former group withdrew consent for immediate postoperative follow-up, while in one patient in the ropivacaine group there was prolongation of the operation and intraoperative modification of the surgical plan. Therefore, data regarding the immediate postoperative period were collected and analyzed in 38 patients of the DR group and 39 patients of the R group. Two additional patients in the DR group and three additional patients in the R group could not be contacted via phone for information regarding chronic pain at 6 and 12 months postoperatively; therefore, data in this respect were collected for 36 patients in each group. The flowchart of the study with patient recruitment, allocation, follow up and analysis is presented in Figure 1.

Patient demographics and the duration of surgery were similar in the two groups (Table 1). Patients in the DR group required less remifentanil intraoperatively as compared to those in the R group (0 [0–0] vs. 0.55 [0–2.65] mcg/kg), *p* < 0.001). The DR group also had a lower requirement for morphine boluses during PACU stay as compared to the R group (0 [0–1] vs. 0 [0–2] mg, *p* = 0.012). Median morphine consumption via the PCA device in the first 24 h was also lower in the DR group as compared to the R group (0 [0–2] vs. 2 [0–2] mg, *p* < 0.001). Consequently, overall morphine consumption postoperatively in the DR group was lower than in the R group (0 [0–2] vs. 3 [0–4] mg, *p* < 0.001). In the early postoperative period during patient PACU stay, NRS scores were significantly different only 60 min post-surgery, with the DR group demonstrating lower scores as compared to the R group (3 [2,3] vs. 3 [3,4], *p* = 0.006). At 5, 15 and 30 min after the operation, no differences in NRS scores were demonstrated between the two groups (*p* = 0.232, 0.164 and 0.091, respectively) (Table 2).

Moreover, patients who were administered the dexmedetomidine–ropivacaine mixture demonstrated significantly less pain at rest 3, 6 and 12 h postoperatively as compared to patients who were administered ropivacaine only (*p =* 0.002, 0.032 and 0.049, respectively). Significant differences between the two groups were also noted for NRS scores during coughing at 3, 6 and 12 h postoperatively (*p* = 0.013, 0.035 and 0.042, respectively. By 24 h post-surgery, NRS scores at rest and during coughing were no longer different between the two groups (*p* = 0.639 and 0.349 for NRS at rest and during coughing, respectively) (Table 2).

Incidence of hypotension and bradycardia as well as sedation scores were no different between the two groups. Similarly, patient satisfaction from anesthesia did not differ between the two groups, while there was no difference in the incidence of PONV or the request for antiemetics (Table 2). Additionally, the incidence of complications related to the performance of the TAP block was low and comparable between the two groups. In specific, one patient in the RD group complained of hypesthesia of the inner thigh region, which was self-limiting in nature and resolved completely within a week, and one patient in the R group developed localized bruising at the site of block performance without signs of infection that resolved within a couple of days.

Six months after the operation, the incidence of chronic pain or abnormal sensation at the operation site was lower in the group that received dexmedetomidine as an adjuvant, with a lower percentage of patients reporting pain at the surgical area versus the ropivacaine-only group (5.55% vs. 25%, *p* = 0.049). By 12 months after surgery, pain or abnormal sensation at the operation site was no different between the two groups (2.77% vs. 8.33%, *p* = 0.614) (Table 2).

## 4. Discussion

According to the results of this randomized double-blinded controlled trial performed in the context of inguinal hernia repair, patients subjected to ultrasound-guided TAP block with a combination of ropivacaine and dexmedetomidine required a lower dose of intraoperative remifentanil infusion, had a lower need for supplementary analgesia in the PACU and consumed less morphine postoperatively via the PCA device than patients subjected to TAP block with ropivacaine alone. Additionally, NRS scores in the former group were significantly lower in the period starting 60 min post-surgery up to 12 h postoperatively. There was not an increased incidence of side effects such as sedation and bradycardia in the ropivacaine–dexmedetomidine group. There was also evidence of a reduced incidence of chronic postoperative pain six months post-surgery when the local anesthetic was supplemented with dexmedetomidine.

The TAP block consists of injecting local anesthetic into the plane between the internal oblique and the transversus abdominis muscles, aiming to block the sensory innervation of the anterior abdominal wall. It has become increasingly popular in recent years, especially since the advent of ultrasound, which greatly increases the efficacy of the technique in comparison to the blind technique using anatomical landmarks [28]. Ultrasound guidance enables precise visual assessment of drug injection and allows the safe spread of the local anesthetic solution in the correct plane between the internal oblique and transversus abdominis fascia layers, close to the targeted nerves [14]. It has been shown to provide better pain relief than placebo, a fact also confirmed in various metanalyses [4,5,6,14]. Additionally, according to a recent metanalysis, it provides superior analgesia as compared to would infiltration up to the first 12 postoperative hours as well as reduced opioid consumption up to 24 postoperative hours [15].

Since the TAP block, unlike other peripheral nerve blocks, is a field block requiring a large volume of local anesthetics to cover several spinal nerves, further prolongation of the blockade duration by increasing the dose of local anesthetic is not always feasible due to local anesthetic-associated potential toxicity [8]. In the quest for prolonging the duration of action of the TAP block, various adjuvants have been investigated in recent years, with occasionally conflicting results. For example, in the context of TAP block for inguinal hernia repair, Kartalov et al. demonstrated that dexamethasone supplementation of the local anesthetic led to decreased postoperative pain scores as well as reduced cumulative 24 h morphine consumption as compared to the local anesthetic alone [29]. However, in another study, supplementation of ropivacaine by dexamethasone in TAP for inguinal hernioplasty did not lead to a statistically significant prolongation of analgesia in comparison to ropivacaine alone [30].

Another adjuvant which has attracted interest in recent years is dexmedetomidine, an α-2 adrenoceptor agonist. Systemically administered dexmedetomidine possesses sympatholytic, sedative and anesthetic-sparing properties [18]. Recently, it has been the subject of increased interest for perineural use in various peripheral regional anesthetic blocks, with the potential to prolong blockade duration [20,21,31]. Likewise, various studies have investigated the addition of dexmedetomidine to the local anesthetic solution to improve the analgesia afforded by the TAP block in various surgical contexts and have documented its favorable effect [32,33,34,35]. Some studies have also documented the superiority of dexmedetomidine as an adjuvant in the TAP block for various surgeries in comparison to other adjuvants [36,37]. Therefore, the investigation of dexmedetomidine as an adjuvant in the context of TAP block for inguinal hernia repair is of interest, especially given the contradictory data available in the literature with other adjuvants in the case of hernioplasty, as mentioned before.

In our study, we demonstrated an intraoperative opioid-sparing effect of the ropivacaine–dexmedetomidine combination, since remifentanil requirements were lower as compared to the ropivacaine-only group. There was also a sustainable analgesic benefit with dexmedetomidine supplementation of the local anesthetic, since patients in the dexmedetomidine–ropivacaine group had lower requirements for supplementary analgesia both in the PACU and in the ward via the PCA device and also experienced less pain up to 12 h postoperatively. The fact that the benefit of the combination became obvious only 60 min after the operation may be due to the fact that at the initial postoperative stages the effect of the ropivacaine was still present, but as soon as its action started to wear off, the presence of dexmedetomidine ensured a sustained analgesic benefit, exceeding the duration of action of the local anesthetic.

There are many theories as to how dexmedetomidine administered perineurally augments the effect of local anesthetics. At the peripheral level, it has been postulated that dexmedetomidine maintains the hyperpolarized state of cells and impedes subsequent action potentials by blocking potassium channels, thus amplifying the effect of local anesthetics [38]. Additionally, by activating α2 adrenoceptors in the peripheral blood vessels, it can constrict these vessels around the injection site, delaying the absorption of local anesthetics and thus pronging blockade duration [39]. At the spinal cord level, dexmedetomidine can suppress neuronal firing via reduction in the release of excitatory neurotransmitters such as glutamate and substance P in the spinal dorsal horn by binding to local α2 adrenoceptors [40]. Additionally, increased hyperpolarization of postsynaptic dorsal horn neurons suppresses the ascending spinal pathway related to nociceptive sensation and contributes to analgesia. Finally, there is evidence that dexmedetomidine can spread to the cerebrospinal fluid via systemic absorption and thus have a central-level effect by acting on α2 receptors in the brainstem and by inhibiting descending noradrenergic pain signals. In fact, El Sherif et al. showed that systemic absorption of dexmedetomidine administered as an adjuvant in TAP block is common [41]. There is also evidence of additional interactions between dexmedetomidine and local anesthetics, which further support its use as an adjuvant. Dexmedetomidine has been shown to attenuate the convulsive potency of both bupivacaine and levobupivacaine in rats [22]. Dexmedetomidine-afforded neuroprotection and reduction in local anesthetic neurotoxicity via various cellular mechanisms has also been demonstrated [23,24,25].

To our awareness, there is paucity of literature on the combination of ropivacaine and dexmedetomidine for TAP block in the context of inguinal hernia repair in the adult population. There is only one pediatric study, in which the authors investigated the efficacy of a reduced-dose 0.2% ropivacaine–dexmedetomidine combination compared to 0.375% ropivacaine in ultrasound-guided TAP block [42]. In accordance with our findings, they demonstrated that the combination group exhibited a significantly longer duration of postoperative analgesia, as shown by an increased time to first rescue analgesia and lower pain scores as compared to ropivacaine alone. After performing an extensive literature search, we could identify only two studies with the use of dexmedetomidine as an adjuvant for the TAP block in the context of inguinal herniorrhaphy in the adult population. However, these studies deal with different local anesthetics. In the first study, Madangopal et al. evaluated the addition of two different doses of dexmedetomidine (0.25 mcg/kg and 0.5 mcg/kg) as an adjuvant to bupivacaine 0.25% [26]. They concluded that the addition of dexmedetomidine 0.5 mcg/kg prolongs the time to the first analgesic request, reduces total consumption of diclofenac postoperatively and leads to lower pain scores as compared to TAP block with bupivacaine alone or TAP block with bupivacaine and dexmedetomidine 0.25 mcg/kg. Therefore, they suggested that dexmedetomidine at a dose of 0.5 mcg/kg is better than the dose of 0.25 mcg/kg as an adjuvant to 0.25% bupivacaine. In a similar study, Talebi et al. performed TAP block with bupivacaine 0.125% that was supplemented with 0.5, 1 or 1.5 mcg/kg of dexmedetomidine [27]. They demonstrated that the maximum duration of the block was longer, the need for supplemental analgesics was lower and patients experienced less pain with the two higher doses of dexmedetomidine. However, based on the higher postoperative sedation scores with the higher dexmedetomidine doses, they recommended 1 mcg/kg as the optimal dose of supplemental dexmedetomidine.

The novelty of our study lies not only in the fact that we used ropivacaine, which has a superior safety profile in comparison to bupivacaine, which was used in the two aforementioned studies, but also in the fact that in those studies the operation took place under spinal anesthesia and the TAP block was performed after the end of surgery. Given the fact that our patients were operated under general anesthesia, we performed the TAP block before the start of the operation, since our goal was to take advantage of the antinociceptive potential of the regional technique not only postoperatively but also intraoperatively, a fact which was corroborated by our findings. Based on the aforementioned literature, we selected the dose of 0.5 mcg/kg for dexmedetomidine, given the reports of higher sedation scores and bradycardia with higher doses [27,43]. As we demonstrated in our study, with the dose of 0.5 mcg/kg that we selected for dexmedetomidine supplementation, the incidence of bradycardia and sedation in both groups was low and was not affected by the presence of dexmedetomidine. Therefore, a dose of 0.5 mcg/kg seems to provide an optimal balance between adequate postoperative analgesia and the occurrence of adverse effects.

An additional interesting finding of our study is the fact that despite the difference in NRS scores and the increased request for supplementary analgesia via the PCA device in the ropivacaine-only group, satisfaction from analgesia was similar in the two groups. It has been documented in the literature that when patients rely on a PCA device for provision of analgesia when it is needed, a sense of self-sufficiency and independence is established, which, together with the perception of being in control, leads to increased satisfaction from pain management [44,45].

The incidence of chronic pain with local anesthetic-only TAP block demonstrated in our study is in accordance with previous reports [46,47]. Chronic pain after inguinal hernia repair can be multifactorial in origin and can adversely affect the quality of life after the operation. Although the precise mechanism leading to chronic pain remains elusive, inadequate management of acute postoperative pain can be one of the leading causes implicated in its development [3]. According to the current literature, the performance of the TAP block may have variable or contradictory preventive effects on the occurrence of chronic pain after inguinal hernia repair [47,48].

We demonstrated that dexmedetomidine contributed to a decreased incidence of chronic pain six months after the operation. To our awareness, this is the first study examining the effect of dexmedetomidine on chronic pain after TAP block for herniorrhaphy. Our literature search was scarce regarding the effect of perineurally administered dexmedetomidine on chronic pain after peripheral nerve blocks. In fact, we identified only two studies, in a different setting, where dexmedetomidine had a favorable effect on the occurrence of chronic pain when it was administered as an adjuvant to erector spinae plane blocks for video-assisted thoracic surgery [49,50]. If we hypothesize that chronic pain is associated with a degree of sympathetic hyperactivity, the inhibition of the release of norepinephrine by dexmedetomidine could result in partial attenuation of this hyperactivity [51]. There is also experimental evidence that dexmedetomidine is able to suppress proinflammatory cytokine release and attenuate glial cell activation, thus helping to alleviate the inflammatory component of chronic pain [52]. Finally, the perineural administration of dexmedetomidine could exert a degree of central and peripheral neuroprotective effects via antioxidative action, the inhibition of apoptosis and the promotion of neurogenesis [53]. All the aforementioned mechanisms could contribute to the reduction in the incidence of chronic pain afforded by dexmedetomidine. Despite the indications of a better outcome with dexmedetomidine regarding the incidence of chronic pain by our findings, we should, however, bear in mind that our study was not powered to the occurrence of chronic pain. Given the scarcity of clinical data on this subject, the purported advantages of dexmedetomidine on chronic pain when administered as an adjuvant in peripheral nerve blocks should be substantiated in future studies adequately powered to the development of chronic pain, which is an outcome requiring a much higher number of patients.

The incidence of complications was low in our study. Performance of TAP block under ultrasound guidance allows the accurate deposition of local anesthetic in the correct neurofascial plane, thus minimizing procedure-related complications. Finally, as mentioned before, the incidence of PONV, sedation and bradycardia was also low, probably due to the fact that we used a low dose of dexmedetomidine. However, we should note that our study was not powered to the detection of side-effects. Still, our results are in accordance with a metanalysis examining the addition of dexmedetomidine in TAP block for various abdominal surgeries, which also demonstrated no effect of dexmedetomidine on the aforementioned outcomes [35].

## 5. Limitations

Our study has certain limitations. We did not measure dexmedetomidine plasma concentration; therefore, we cannot be certain whether its action is due to systemic absorption or a local effect. Our patients were not followed up after 24 h, so our results are valid for the first postoperative day only. Finally, we only included ASA I and II patients, so the efficacy and safety of dexmedetomidine in the cohort of high-risk patients remains uncertain.

## 6. Conclusions

Under the present study design, we demonstrated that dexmedetomidine as an adjuvant to ropivacaine prolonged the duration of analgesia, reduced postoperative pain scores, reduced analgesic intake and had a favorable effect on chronic pain without increasing postoperative complications in patients subjected to TAP block as a supplementary analgesic modality for inguinal hernia repair.

## Figures and Tables

**Table 1:** Demographic characteristics of the dexmedetomidine–ropivacaine (DR) and ropivacaine (R) groups.

Variables	DR Group (n = 38)	R Group (n = 39)	*p* Value Between Groups
**Age (years)**	63.3 ± 11.9	61.7 ± 15.1	0.609 ^#^
**Gender (m/f)**	34/4	35/4	1.000 ^$^
**Weight (kg)**	78.2 ± 9.3	74.4 ± 8.5	0.062 ^#^
**Height (cm)**	168.6 ± 7.4	171.0 ± 7.0	0.144 ^#^
**ASA (I/II)**	26/12	24/15	0.694 ^%^
**Duration of surgery (min)**	76.8 ± 16.7	72.0 ± 15.1	0.192 ^#^

Data are presented as mean ± SD, absolute number or median [25th–75th percentile]; ^#^ unpaired *t*-test; ^$^ Fisher exact test; ^%^ Chi square test; ASA, American Society of Anesthesiologists.

**Table 2:** Pain parameters and NRS scores of the dexmedetomidine–ropivacaine (DR) and ropivacaine (R) groups.

Variables	DR Group (n = 38)	R Group (n = 39)	*p* Value Between Groups
**Intraoperative remifentanil consumption (mcg kg^−1^)**	0 [0–0]	0.55 [0–2.65] *	**<0.001** ^^^
**Morphine consumption (mg) (PACU)**	0 [0–1]	0 [0–2] *	**0.012** ^^^
**Morphine consumption (mg) (PCA)**	0 [0–2]	2 [0–2] *	**<0.001** ^^^
**Overall morphine consumption (mg) postoperatively**	0 [0–2]	3 [0–4] *	**<0.001** ^^^
**NRS (rest) during PACU stay**			
5 min	2 [1–3]	2 [2–3]	0.232 ^&^
15 min	2 [2–3]	3 [2–3]	0.164 ^&^
30 min	2 [2–3]	3 [2–3]	0.091 ^&^
60 min	3 [2–3]	3 [3–4] *	**0.006** ^&^
**Postoperative NRS (rest)**			
3 h	2.5 [2–3]	3 [3–3] *	**0.002** ^&^
6 h	3 [2–3]	3 [2–4] *	**0.032** ^&^
12 h	2 [2–3]	3 [2–3] *	**0.049** ^&^
24 h	2 [1–2]	2 [2–2]	0.639 ^&^
**Postoperative NRS (movement)**			
3 h	3 [3–3]	3 [3–4] *	**0.013** ^&^
6 h	3 [3–3]	3.5 [3–5] *	**0.035** ^&^
12 h	3 [2–3]	3 [3–5] *	**0.042** ^&^
24 h	2 [2–2]	2 [2–3]	0.349 ^&^
**Incidence of hypotension, number of patients**	7	5	0.716 ^%^
**Incidence of bradycardia, number of patients**	3	3	1.000 ^$^
**Sedation score**	1 [1–2]	1 [1–2]	0.550 ^^^
**Patient satisfaction, Likert scale**	4 [3–4]	4 [3–4]	0.726 ^^^
**PONV incidence, number of patients**	2	1	0.615 ^$^
**Request for ondansetron, number of patients**	1	1	1.000 ^$^
**Pain or abnormal sensation 6 months postoperatively, number of patients**	2/36	9/36 *	**0.049** ^%^
**Pain or abnormal sensation 12 months postoperatively, number of patients**	1/36	3/36	0.614 ^$^

* Significant difference between groups; ^$^ Fisher exact test; ^%^ Chi square test; ^^^ Mann–Whitney U-test; ^&^ two-factor mixed-design ANOVA with Student–Neuman–Keuls test for individual differences; **bold** in *p* values for significant differences; data are presented as absolute numbers or as median [25th–75th percentile]. PACU, Post Anesthesia Care Unit; PCA, Patient Control Analgesia; NRS, numeric rating scale; PONV, postoperative nausea and/or vomiting.

## Data Availability

The datasets generated during and/or analyzed during the current study are available from the corresponding author on reasonable request.

## Supplementary Materials

The following supporting information can be downloaded at https://www.mdpi.com/article/10.3390/jcm14072478/s1, Video S1: sonographic video depicting the performance of the TAP block.
